# The complete mitochondrial genome of the causative agent of the human cercarial dermatitis, the visceral bird schistosome species *Trichobilharzia szidati* (platyhelminthes: Trematoda: Schistosomatidae)

**DOI:** 10.1080/23802359.2017.1347833

**Published:** 2017-08-02

**Authors:** Seraphima Semyenova, Galina Chrisanfova, Ludmila Mozharovskaya, Andrei Guliaev, Alexey Ryskov

**Affiliations:** Laboratory of Genome Organization, Institute of Gene Biology of the Russian Academy of Sciences, Moscow, Russia

**Keywords:** Mitochondrial genome, cercarial dermatitis, trematoda, bird schistosomes, phylogenetic relationships

## Abstract

We report the first complete mitochondrial genome of visceral bird schistosome *Trichobilharzia szidati* **(**Platyhelminthes, Trematoda: Schistosomatidae). The circular genome is 14293 bp in length and contains 12 protein-coding genes, 12S and 16S rRNAs genes, 22 tRNAs and one non-coding region (202 bp) (accession number MF136777). Phylogenetic relationships based on 12 protein-coding gene sequences (PCG) of mitogenomes of a number of trematode and cestode species have shown that *T. szidati* is the closest genetic relative to nasal bird schistosome *T. regenti.* The complete mitogenome sequence of *T. szidati* may serve as a resource for comparative mitogenomics and trematode evolution studies**.**

Zoonotic disease cercarial dermatitis (swimmer’s itch) occurs when larval stages of bird schistosomes that normally develop in birds penetrate human skin. The majority of bird schistosomes *Trichobilharzia* spp. including *T. szidati* live in the blood system of visceral organs, only mature *T. regenti* occurs in the nasal mucosa of its definitive host (Horák and Kolářová 2011). The complete mitochondrial genome is known only for nasal representative of bird schistosomes (Webster et al. 2007).

We report for the first time the complete sequence of mitogenome of visceral species *T. szidati* and revise its phylogenetic position. It will be useful for the studies in population genetics, comparative mitogenomics, phylogenetics and evolutionary studies of trematodes.

 Cercariae of *T. szidati* were collected from host snail *Lymnaea stagnalis* from Lake Naroch (54°54′24.1848″N, 26°42′ 14.3532″E) in Belarus. The specimen (N15Lst1) is stored in the Laboratory of Genome Organization, Institute of Gene Biology of the Russian Academy of Sciences, Moscow, Russia. Total genomic DNA was extracted by NEB #E6000 Kit (Illumina, Inc, San Diego, CA) following the instructions of the manufacturer. DNA sequencing was conducted using Illumina Hiseq 2000 (Illumina, Inc, San Diego, CA).

Library preparation, DNA sequencing, mitogenome assembling using MITObim (Hahn et al. 2013) were performed at the Genoanalityka JSC, Moscow. The mitogenome annotation was conducted via BLAST (Altschul et al. 1990) against mitogenome of *T. regenti* (DQ859919). Phylogenetic position of *T. szidati* was estimated on the base of protein-coding genes (PCG) of the mitogenomes of platyhelminth species from class Trematoda: Schistosomatidae: *T. regent Schistosoma haematobium*,* S. mansoni*,* S. japonicum*,* S. mekongi*; Paragonimidae: *Paragonimus westermani*; Fasciolidae: *Fasciola gigantica* and *F. hepatica* and two species from class Cestoda: *Diphyllobothrium latum* and *Taenia solium* (as an out group). We constructed a phylogenetic tree using PhyML (Guindon et al. 2010) and MrBayes (Ronquist and Huelsenbeck 2003).

The complete mtDNA sequence of *T. szidati* is 14,293 bp in length, which is slightly larger than the mitogenomes of two Asiatic mammalian flukes, *S. Mekongi* (14,072 bp) and *S. japonicum* (14,085 bp), but smaller than mitogenomes of two African flukes *S. mansoni* (14,415 bp), *S. haematobium* (15,003 bp) and nasal schistosome *T. regenti* (14,838 bp).

The gene order of *T. szidati* mitochondrial genome is identical to *T. regenti* and Asiatic mammalian schistosomes described earlier (Webster and Littlewood 2012) and contains 12 PCG, 22 transfer RNA genes and two ribosomal RNA genes. The size of the PCG of the *T. szidati* and *T. regenti* did not vary, except of *nad3* (360 bp vs. 363bp), *nad5* (11590 bp vs. nd 1599 bp) and *cytb* (1107 bp vs. 1110 bp). The overall sequence difference between concatenated PCG of *T. regenti* and *T. szidati* is 17.6%.

In both *Trichobilharzia* species, the genes of two mitochondrial rRNA subunits, *rrnL* and *rrnS*, are separated by *trnC*. The mitogenome of *T. szidati* includes a non-coding region (202 bp) with two similar short sequences (25 bp). In contrast, the long non-coding region in *T. regenti* includes three tandem repeats of 184 bp each.

The phylogenetic study provides a strongly supported framework for the monophyly of the Schistosomatidae subgroup and supports that the *T. szidati* is closely related to *T. regenti* (Figure 1).

**Figure 1.**
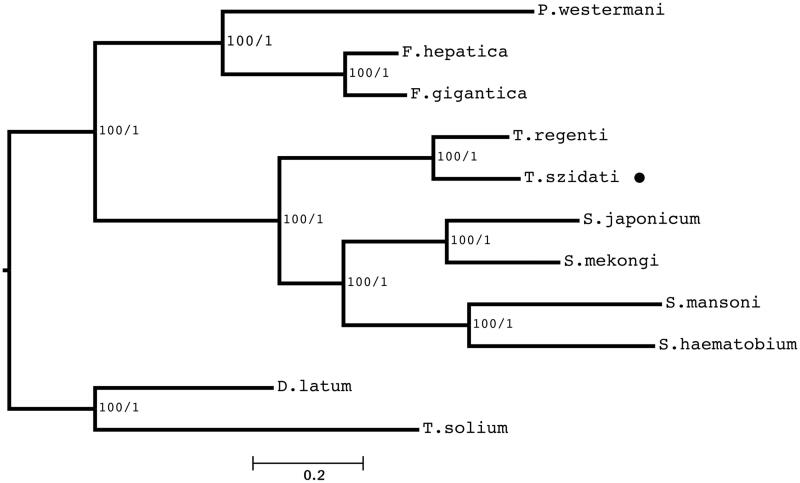
The phylogenetic relationship of nine trematode species, including *T. szidati* (marked with black circle)**, **based on the nucleotide sequences of 12 PCGs. ML bootstrap values and Bayesian posterior probabilities are shown at the nodes of the tree. The species names and GenBank accession numbers provided as follows: *Trichobilharzia regenti,* DQ859919.1; *Schistosoma mansoni,* AF216698; *Schistosoma japonicum*, AF215860; *Schistosoma mekongi,* AF217449; *Schistosoma haematobium*, DQ157222; *Paragonimus westermani*, NC_002354.2; *Fasciola gigantica*, KF543342.1; *Fasciola hepatica*, NC_002546; *Taenia solium*, AB086256.1; *Diphyllobothrium latum*, AB269325.1.

## References

[CIT0001] Altschul SF, Gish W, Miller W, Myers EW, Lipman DJ. 1990 Basic local alignment search tool. J Mol Biol. 215:403–410. 2231712 10.1016/S0022-2836(05)80360-2

[CIT0002] Guindon S, Dufayard JF, Lefort V, Anisimova M, Hordijk W, Gascuel O. 2010 New algorithms and methods to estimate maximum-likelihood phylogenies: assessing the performance of PhyML 3.0. Syst Biol. 59:307–321. 20525638 10.1093/sysbio/syq010

[CIT0003] Hahn C, Bachmann L, Chevreux B. 2013 Reconstructing mitochondrial genomes directly from genomic next-generation sequencing reads-a baiting and iterative mapping approach. Nucleic Acids Res. 41:e129. 23661685 10.1093/nar/gkt371 PMC3711436

[CIT0004] Horák P, Kolářová L. 2011 Snails, waterfowl and cercarial dermatitis. Freshwater Biol. 56:779–790.

[CIT0005] Ronquist F, Huelsenbeck JP. 2003 MrBayes 3: Bayesian phylogenetic inference under mixed models. Bioinformatics. 19:1572–1574. 12912839 10.1093/bioinformatics/btg180

[CIT0006] Webster BL, Littlewood DT. 2012 Mitochondrial gene order change in Schistosoma (Platyhelminthes: Digenea: Schistosomatidae). Int J Parasitol. 42:313–321. 23362512 10.1016/j.ijpara.2012.02.001

[CIT0007] Webster BL, Rudolfová J, Horák P, Littlewood DT. 2007 The complete mitochondrial genome of the bird schistosome *Trichobilharzia regenti* (Platyhelminthes: Digenea), causative agent of cercarial dermatitis. J Parasitol. 93:553–561. 17626347 10.1645/GE-1072R.1

